# Investor taxation, firm heterogeneity and capital structure choice

**DOI:** 10.1007/s10797-019-09536-x

**Published:** 2019-04-09

**Authors:** Silke Rünger, Rainer Niemann, Magdalena Haring

**Affiliations:** Institute of Accounting and Taxation, University of Graz, Universitätsstraße 15, 8010 Graz, Austria

**Keywords:** Capital structure, Investor taxation, Ownership, Dividend payout policy, G32, H24, H25, H32

## Abstract

In this paper, we analyze the effect of investor level taxes, firm-specific ownership structure and firm-specific dividend payout policy on a firm’s capital structure choice. Our analysis is based on data for 10,003 firms from 11 Central and Eastern European (CEE) countries over the period 2002–2012. Our results show a significant positive impact from the net tax benefit of debt on the debt ratio of a firm. Ignoring firm heterogeneity, an increase in the net tax benefit of debt by 10 percentage points leads to an increase in the debt ratio of 2.68 percentage points. If we add firm-specific ownership to the analysis, the effect of investor level taxes on the debt ratio is about 1.55 times higher if the firm is wholly owned by a domestic individual investor. For the same type of firm, the effect nearly doubles if we also consider firm-specific dividend payout policy.

## Introduction

Since Modigliani and Miller (1958), the effect of taxes on the capital structure of a firm has been under ongoing investigation. From a theoretical standpoint, Miller (1977) showed that both corporate and investor level taxes must be considered in capital structure choices. The benefit arising from interest deductibility at the corporate level must be weighed against the so-called personal tax penalty. A personal tax penalty occurs at the investor level because personal income tax on interest income from debt is often higher than the personal income tax on equity income (dividends and capital gains).

In this paper, we analyze the effect of investor level taxes on capital structure choice and control for firm heterogeneity using firm-specific ownership structure and firm-specific dividend payout policy. Prior research has identified many different sources of firm heterogeneity, such as firm size, firm age or firm profitability, which affect capital structure choice. We, however, focus upon ownership and dividend payout policy as they are pivotal sources of firm heterogeneity that affect investor level taxes.

We add to prior literature by providing a more precise estimation of the effect of investor level taxes on capital structure choice. First, contrary to prior research, which assumes that the marginal investor of the firm is a domestic individual investor in the highest tax bracket, we directly observe firm-specific ownership on a yearly basis and thus can identify the marginal investor of the firm. Second, all of our sample countries apply constant marginal tax rates on all sources of income relevant to our analysis (that is, corporate, interest, dividend, and capital gains income). Our setting allows us to calculate the investor level tax burden without the need for investor-specific information such as the level of other income or holding periods. Third, by considering the firm-specific dividend payout policy, we can account for the fact that corporate profits can either be distributed to owners as dividends or retained to increase firm value, which leads to future capital gains.

We use the Gordon and MacKie-Mason (1990) and Graham (1999) definition of the net tax benefit of debt to test the effect of investor level taxes on capital structure choice. Our sample consists of 10,003 private firms from 11 CEE countries over the period 2002–2012. We restrict our analysis to countries that apply constant marginal tax rates. If we consider investor level taxes and do not control for firm heterogeneity, we find an increase in the net tax benefit of debt of 10 percentage points to lead to an increase in debt ratios by 2.68 percentage points. When looking at the tax rates individually, we find that corporate taxes as well as dividend and capital gains taxes have a significant positive impact and the interest tax rate has a significant negative impact on debt ratios. We calculate elasticities to assess the economic significance of our results and are able to show that taxes are as important as other traditional variables in explaining debt ratios.

Pindado and de la Torre (2011) argue that the capital structure of a firm is determined by the incentives of those investors who are in control of the firm. We therefore add to our estimation information on firm-specific ownership. We show that the effect of investor level taxes on debt ratios increases if we consider firm-specific ownership, consistent with a reduction in measurement error. We attribute our findings to two main reasons. Firstly, there is a natural link between firm-specific ownership and investor level taxes. Investor level tax rates, which are needed for the calculation of the net tax benefit of debt, depend on the investor who is in control of the firm, also called the marginal investor of the firm. Depending upon whether this marginal investor is a fully taxable individual or a tax-exempt institutional investor substantially changes the personal tax penalty. As our dataset allows us to observe the firm-specific ownership structure and thus the marginal investor of the firm, we are able to show the measurement error that occurs if the marginal investor of a firm is not an individual. Secondly, prior research has shown that agency conflicts between owners might prevent a tax-efficient capital structure, even if all owners face the same tax rates. We address this problem by varying the definition of the marginal owner. Our results show that the effect of the dividend tax rate on debt ratios increases as agency conflicts between owners decrease. We find the opposite effect for the capital gains tax rate, consistent with liquidity restraints on the disposal of large owner blocks.

We expect the firm-specific dividend payout ratio to affect the capital structure of the firm for two reasons. First, King (1977) claims that there is a direct link between capital structure and firm-specific dividend payout policy as retained profits add to the equity capital of the firm. Second, as with firm-specific ownership, there is a natural link between firm-specific dividend payout policy and investor level taxes. Dividends paid to the investor are taxed upon distribution, whereas retained profits add to the equity capital of the firm and thus increase firm value as well as the capital gain that can be realized by the investor when selling shares of the firm. If dividends and capital gains are taxed at different tax rates at the investor level, ignoring capital gains taxation imposes measurement error. Consistent with prior literature, we find that the effect of the dividend tax rate on capital structure is significantly lower if the firm does not pay dividends.

If we consider both sources of firm heterogeneity simultaneously, we do not find an incremental explanatory power of firm heterogeneity for firms without a majority owner. However, if firms are majority-owned by a domestic individual, controlling jointly for firm-specific ownership and firm-specific dividend payout policy increases the effect of the net tax benefit of debt on debt ratios by 38%.

We contribute to prior literature regarding capital structure choice in several ways. First, we show that investor level taxes are as important as other traditional variables in explaining debt ratios in private firms. Second, we enhance prior research by analyzing whether controlling for firm heterogeneity yields a more precise measure of the effect of investor level taxes on debt ratios. Third, we are able to show that there is no general improvement in measurement precision when we consider firm heterogeneity. If the firm’s ownership composition causes high agency costs, we find no incremental effect from firm heterogeneity on the relation between investor level taxes and debt ratio. Conflicting interest of owners mitigates the benefit of a more precise measurement of the net tax benefit of debt.

The remainder of this paper is organized as follows: In Sect. 2, we present the model of the net tax benefit of debt and show how firm heterogeneity can be included in the calculation of the net tax benefit of debt. Furthermore, we derive the hypotheses and describe the institutional background. Our data set and descriptive statistics are shown in Sect. 3. Results of our regression analysis are presented in Sect. 4, robustness checks are conducted in Sect. 5. Section 6 concludes.

## Net tax benefit of debt and institutional background

### Calculation of the net tax benefit of debt including firm heterogeneity

From a corporate perspective, interest payments for debt are tax-deductible and create an interest tax-shield while payments to equity investors are not deductible. As a result, debt is more attractive than equity. Miller (1977) shows that this relationship does not necessarily hold if investor level taxes are taken into account. Investor level taxes might cause a so-called personal tax penalty, if dividends and/or capital gains are taxed at lower tax rates than interest payments. Prior empirical research that analyzes capital structures has used the Miller Tax Index (Faccio and Xu 2015) or a linear version of it (Overesch and Voeller 2010; Babbel et al. 2018) to model the effect of investor level taxes on debt. The linear version of the Miller Tax Index, the so-called net tax benefit of debt, is calculated as follows:

1 $$ \begin{aligned} {\text{NTBD}}   & = \left( { 1 - \tau_{i} } \right) - \left( {1 - \tau_{c} } \right) \cdot \left( {1 - \tau_{d} } \right) \\ & = \tau_{c} + \tau_{d} - \tau_{c} \cdot \tau_{d} - \tau_{i} \\ \end{aligned} $$

In line with results from prior research, our baseline hypothesis reads as follows:

#### **Hypothesis 1**

The debt ratio of a firm is higher, the higher the net tax benefit of debt.

In this paper, we analyze the effect of two sources of firm heterogeneity on the relation between the net tax benefit of debt and debt ratios: firm-specific ownership as well as firm-specific dividend payout policy.

Agency conflicts between majority and minority owners (Shleifer and Vishny 1986) or between owners and management (Jensen and Meckling 1976) might prevent a tax-efficient capital structure for a firm. Subsequently, many studies have shown that the firm-specific ownership structure is likely to be an important determinant of the firm’s capital structure (Brailsford et al. 2002; Miguel et al. 2005; D’Mello and Miranda 2010; Pindado and de la Torre 2011; Pindado et al. 2015; Schulze et al. 2003). Krämer (2015) further extends this result by analyzing how ownership concentration affects the relationship between taxes and debt.

Results from prior research suggest that the capital structure of a firm is determined by the incentives of the investors who are in control of the firm. Pindado and de la Torre (2011) refer to this as the “ownership view of capital structure”. Investor level taxes play a crucial role in correctly modeling the incentives of the owners who are in control of the firm. Therefore, when calculating the net tax benefit of debt as shown in Eq. (1), one must choose which investor level tax rates to use. Prior literature (see, among others, Overesch and Voeller 2010; Lin and Flannery 2013; or Faccio and Xu 2015), uses the tax rates of domestic individual investors belonging to the highest tax bracket. Thus, most prior literature implicitly assumes that the owner in control of a firm, also called the firm’s marginal owner, is a domestic individual investor in the highest tax bracket. However, La Porta et al. (1999) show that ownership composition varies substantially among firms and countries, with 36% of the firms in their sample being widely held and only 30% being family-controlled. For a sufficiently large number of firms, the marginal owner might not be a domestic individual investor who belongs to the highest tax bracket. Hence, using the tax rates of a domestic individual investor who belongs to the highest tax bracket to calculate the net tax benefit of debt induces measurement error.

To tackle the problem of the identification of the marginal owner when using investor level tax rates, several studies proxy for firm-specific ownership (Lin and Flannery 2013; Faccio and Xu 2015) or use data on observed ownership structures (Fossen and Simmler 2016; Babbel et al. 2018). Similar to Babbel et al. (2018), our dataset allows us to directly observe the composition of the firm-specific ownership structure and thus the marginal owner of the firm. Due to the fact that we use the statutory marginal tax rates for individual domestic owners^1^ to calculate the net tax benefit of debt as shown in Eq. (1), we expect the effect of the net tax benefit of debt on debt ratios to be higher, if the marginal owner of the firm is a domestic individual investor. Our hypothesis therefore reads as follows.

#### **Hypothesis 2a**

The effect of the firm-specific net tax benefit of debt on the debt ratio of the firm is higher if the marginal owner of the firm is a domestic individual investor.

Additionally, our ownership data allows us to test the effect of the net tax benefit of debt on debt ratios if we vary the definition of the marginal owner. Jiang et al. (2017) document an ownership structure pecking order that sorts out which ownership structures are likely to have relatively lower agency costs. Their findings show that firms with a single controlling shareholder have the lowest agency costs and firms with single large non-controlling shareholders have the highest agency costs. In a different setting, Jacob and Michaely (2018) show that the tax sensitivity of owners gradually decreases as the number of owners increases and mainly attribute this finding to agency conflicts. We expect the effect of the net tax benefit of debt on debt ratios to be higher, the lower the agency costs of the firm-specific ownership structure and thus test the following hypothesis:

#### **Hypothesis 2b**

The effect of the firm-specific net tax benefit of debt on the debt ratio of the firm is higher if the firm is held by a single domestic individual investor than if the firm is held by a large non-controlling domestic individual investor.

Despite firm-specific ownership, prior literature has identified another source of firm heterogeneity, namely firm-specific dividend payout policy, to have an influence on the capital structure of the firm. As with firm-specific ownership, there is a direct link between the calculation of the net tax benefit of debt as shown in Eq. (1) and firm-specific dividend payout policy. Using the investor level dividend tax rate assumes that all profits of the firm are distributed to investors as dividends. In contrast, King (1977) points out that the dividend payout policy of the firm affects its capital structure as retained profits add to the equity capital of the firm. Consequently, the more earnings are retained, the smaller the effect of dividend taxes on the capital structure. Building on King (1977) and Auerbach (2002) emphasizes that in a setting in which debt, equity and retentions are determined simultaneously, tax effects on leverage depend upon the dividend payout policy of the firm.

Gordon and MacKie-Mason (1990) provide a measure of the investor level tax rate on equity that considers the firm-specific dividend payout policy as well as the amount of retained earnings. In their model, retained earnings increase the firm value and result in a taxable capital gain if owners sell their shares in the future. If firm-specific dividend payout ratios and the taxation of capital gains are ignored when calculating the net tax benefit of debt, this induces another potential source of measurement error.

One stream of prior literature (e.g., Overesch and Voeller 2010; Faccio and Xu 2015) considers the effect of investor level capital gains taxes, but does not consider firm-specific dividend payout policy. Another section of prior literature considers firm-specific dividend payout policy, but does not interact firm-specific dividend payout policy with investor level tax rates (e.g., Alworth and Arachi 2001; Babbel et al. 2018; Cheng and Green 2008; Givoly et al. 1992). A third section of prior literature uses changes in investor level tax rates around specific tax reforms as a triggering event and controls for different reactions of a firm to the tax rate change based upon the firm-specific dividend payout policy (e.g., Campello 2001; Faccio and Xu 2015; Lin and Flannery 2013; Schulman et al. 1996).

To account for the firm-specific dividend payout policy, we use the Gordon and MacKie-Mason (1990) model of the net tax benefit of debt. In this model, if a firm decides to raise an additional dollar of equity, the firm’s owners receive ($1 - \tau_{c}$)·($1 - \tau_{e}$) of the profits, with $\tau_{e}$ as the tax rate on income from equity. The tax rate on equity income ($\tau_{e}$) can be further decomposed to $\tau_{e}$ = *d*·$\tau_{d}$ + (1 − *d*)·*α*·$\tau_{g}$, where *d* is the firm-specific dividend payout ratio, *α* is a discount factor described below and $\tau_{d}$ and $\tau_{g}$ are the investor level tax rates on dividends and capital gains. The net tax benefit of debt (NTBD_Payout_), considering the firm-specific dividend payout ratio, is calculated as:

2 $$ {\text{NTBD}}_{\text{Payout}} = \left( {1 - \tau_{i} } \right) - \left( {1 - \tau_{c} } \right) \cdot \left( {1 - \left( {d \cdot \tau_{d} + \left( {1 - d} \right) \cdot \alpha \cdot \tau_{g} } \right)} \right) $$

In most countries, capital gains are taxed at tax rates that are different from tax rates on ordinary income. Several countries do not tax capital gains, or tax them at a reduced rate, if some preconditions (minimum thresholds, holding periods) are met. In general, this causes difficulties in determining investor level marginal tax rates on capital gains. All of our sample countries apply a constant marginal tax rate for capital gains, and capital gains are taxed regardless of minimum thresholds or holding periods. We are therefore able to use the statutory marginal tax rate on capital gains and do not need to adjust for investor- or transaction-specific factors.

One important aspect of capital gains taxation is that owners can either defer the capital gains tax payment by deferring the disposal of the asset, or completely avoid capital gains taxation by refraining from selling the shares until death. This avoidance behavior causes a distortional effect known as the lock-in effect of capital gains taxation (Holt and Shelton 1961; Sprinkel and West 1962). To consider an owner’s ability to defer capital gains taxation, we use an effective capital gains tax rate rather than the statutory marginal tax rate on capital gains in our model. We calculate the effective capital gains tax rate as $\tau_{g}^{\text{eff}}$ = *α*·$\tau_{g}$, *α* representing the discounting effect. Feldstein and Summers (1979), Gordon and MacKie-Mason (1990) and Graham (1999) assume *α* to be 0.25. The value of *α* is determined by the discount rate *r* and the expected holding period *T* of the investor as

3 $$ \alpha = \frac{1}{{(1 + r)^{T} }} $$

and decreases if one of the two determinants increases. We assume discount rates to be lower during our observation period than at the time of Feldstein and Summers (1979), Gordon and MacKie-Mason (1990) and Graham (1999) and therefore assume *α* = 0.5, which is consistent with more recent levels of interest rates. We additionally vary the level of *α* in our robustness checks.

Differentiating NTBD_Payout_ with respect to the firm-specific payout ratio *d* yields:

4 $$ \frac{{\partial {\text{NTBD}}}}{\partial d} = \left( {1 - \tau_{c} } \right) \cdot \left( {\tau_{d} - \alpha \tau_{g} } \right)\begin{array}{*{20}c} > \\ { = } \\ < \\ \end{array} 0 $$

This partial derivative has an ambiguous algebraic sign, because the statutory tax rates on dividends and capital gains often differ and because capital gains are taxed upon realization. Although the direction is not specified, the dividend payout ratio affects the net tax benefit of debt. We therefore do not formulate a direct hypothesis but include firm-specific payout ratios into our analysis.

Many studies refrain from integrating firm-specific dividend payout ratios in capital structure choices as there is conflicting empirical evidence that payout policy itself reacts to changes in taxation. Chetty and Saez (2005) and Jacob and Jacob (2014) document that firms adjust their payout policy after tax rate changes, whereas Korkeamaki et al. (2010) and Renneboog and Trojanowski (2011) do not find evidence of dividend clientele effects in single country analysis. Studies showing that payout policy reacts to changes in taxation focus on public firms and compare dividends to share repurchases. Share repurchases might be less important in private firms. Michaely and Roberts (2012) and Jacob and Michaely (2018) show that a potential channel for taxes to influence dividend policy differentially across public and private firms is through differences in owners’ abilities to substitute between dividends and wages rather than dividends and share repurchases. Also, dividend policy of public and private firms is found to differ substantially. Michaely and Roberts (2012) show that dividend payout ratios of public firms are about twice as high as dividend payout ratios in private firms. Moreover, public firms are significantly more averse to omitting or cutting dividends than private firms and wholly owned firms’ dividends are more sensitive to investment needs than those of public firms.

Prior capital structure research investigating the influence of taxation has focused on the net tax benefit of debt as shown in Eq. (1) and assumed that the marginal investor of the firm is a domestic individual in the highest tax bracket and that all profits of the firm are distributed as dividends to the investor. We add information on firm-specific ownership and firm-specific dividend payout policy to the calculation of the net tax benefit of debt to obtain a more precise measurement of the effect of investor level taxes on capital structure choice. A more precise measurement requires (1) the correct identification of the firm-specific marginal investor, (2) the use of the tax rates applicable to the identified marginal investor and (3) a correct weight of the tax rates applicable to the identified marginal investor by using the firm-specific dividend payout policy. A joint examination of these factors yields a more precise estimation of the effect of investor level taxes on debt ratios.

### Institutional background

To avoid measurement error caused by progressive tax schemes, we require our sample countries to apply a constant marginal tax rate for all types of income included in the analysis (corporate income, interest, dividends and capital gains) over the sample period 2002–2012. Among all European countries, we find 11 countries (Bulgaria, Croatia, Estonia, Hungary, Latvia, Lithuania, Poland, Romania, Russia, Slovakia and Ukraine) that have constant marginal tax rates over the sample period. All of our sample countries are located in CEE.^2^ For each country, we collect information on tax rates over the period 2002–2012 using the European Tax Handbooks provided by the IBFD. We calculated the net tax benefit of debt for individual owners and therefore use the tax rate on interest, dividends and capital gains applicable to domestic individuals. The range of tax rates over the observation period is depicted in Table 1.

**Table 1:** Overview of tax rates, 2002–2012

Country	$\tau_{c}$ (%)	$\tau_{i}$ (%)	$\tau_{d}$ (%)	$\tau_{g}$ (%)
Bulgaria	10–20	0–20	5–15	10–29
Croatia	20	0	0–15	0
Estonia	0	21–26	21–26	21–26
Hungary	10–18	0–20	16–25	10–25
Latvia	15–25	10–25	0–10	15–23
Lithuania	15–20	15	15–20	15
Poland	19–28	19–20	15–19	19
Romania	16–25	1–16	5–16	1–16
Russia	20–35	13	6–9	0–13
Slovakia	19–25	19	0–15	19
Ukraine	21–25	5–15	5–15	13–15

This table shows the range of tax rates on corporate profits, $\tau_{c}$, interest income received by individual domestic owners, $\tau_{i} ,$ dividend payments received by individual domestic owners, $\tau_{d} ,$ and capital gains realized by individual domestic owners, $\tau_{g}$, for every sample country over the sample years 2002–2012

All our sample countries, except for Croatia, Estonia and Lithuania, have changed all tax rates used in our model at least once during the observation period. This substantial variation in tax rates creates an ideal setting that can be exploited by our panel data analysis. During the observation period, 9 out of our 11 countries changed their corporate tax rate at least once (16 changes in corporate tax rates altogether). At the investor level, most changes occurred within the taxation of capital gains and dividends (19 changes in dividend tax rates as well as 18 changes in capital gains tax rates in 9 out of the 11 sample countries). We find that taxation of interest income has the lowest variance in tax rates. We identify 15 changes in interest tax rates during the observation period within 9 different countries. It is less important to integrate firm-specific dividend payout policy into the calculation of the net tax benefit of debt, if countries tax dividend payments at the same tax rate as capital gains. In our sample, in 57 out of 121 (47.11%) country-years dividends are taxed at a different rate than capital gains, which marks the importance of considering both tax rates.^3^

**Fig. 1:**
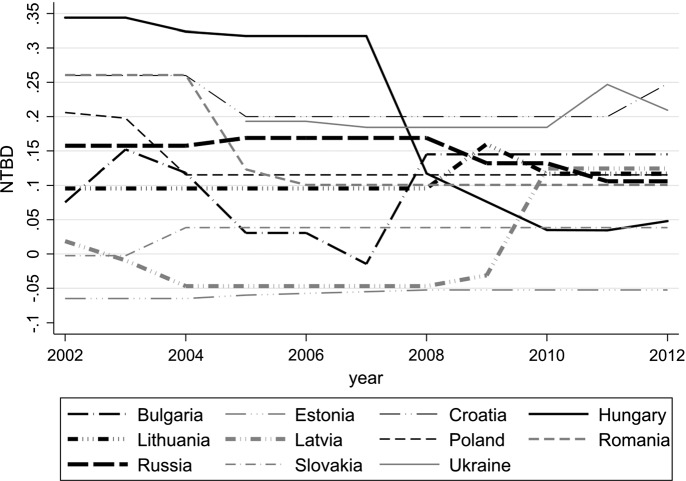
Net tax benefit of debt by country and year, 2002–2012. This figure shows the net tax benefit of debt calculated following the approach of Gordon and MacKie-Mason (1990). Tax rates are taken from Table 1 and results are based on the assumption that half of the firm’s profits are distributed as dividends, thus *d* = 0.5 and *α* = 0.5

Using the tax rates depicted in Table 1, we calculate the country-year-specific net tax benefit of debt. At this stage, instead of using firm-specific dividend payout ratios, we follow Faccio and Xu (2015) and use the blended average of dividend taxes and the effective tax rate on capital gains. To calculate the effective capital gains tax rate, we assume *α* to equal 0.5. Our results are shown in Fig. 1.

Integrating investor level taxes into the calculation of the net tax benefit of debt can cause a personal tax penalty, resulting in an overall negative net tax benefit of debt. Estonia has a negative net tax benefit of debt over the whole observation period. This is due to the fact that in Estonia corporate profits are not taxable until they are distributed to owners, therefore $\tau_{c} = 0$. In addition, investor level tax rates are identical for interest income, dividends and capital gains. In our model, the effective tax rate on capital gains is lower than the statutory tax rate on capital gains due to *α* < 1. Payments to equity holders therefore are more beneficial compared to payments to debt holders when considering investor level taxes. We also find a negative net tax benefit of debt in Latvia during the years 2003–2009, in Slovakia during the years 2002–2003 and in Bulgaria in 2007. Results in Fig. 1 also show that the net tax benefit of debt is converging for many sample countries over the sample period. In 2012, at the end of our observation period, 6 out of the 11 countries have an average net tax benefit of debt in the interval [0.10; 0.15], whereas in 2002, at the beginning of the observation period, we observe a greater variance in the net tax benefit of debt within [− 0.05; 0.35].

## Data and regression model

### Data and summary statistics

We use the AMADEUS database to obtain annual firm-level data (unconsolidated financial reports and ownership information) for private firms in our 11 countries with constant marginal tax rates. As the number of public firms in the sample countries is very low, we decide to stick to private firms only as prior research has shown that financing decisions may differ for private and public firms. In Table 2, we summarize the sample generation process.

**Table 2:** Sample generation process

Description	Firm-year observations	Firms
Financial reports available	90,162	15,036
– Missing data on payout ratio	13,070	3394
– Missing or incomplete ownership data	34,361	1637
– Negative debt ratio	10	2
Final sample (ownership information and full accounting information)	42,721	10,003

This table shows the sample generation process. We start with all firms from 11 CEE countries with financial reports available for at least 1 year (2002–2012) in the AMADEUS database. After dropping firms with missing data on payout ratio and ownership, our main sample consists of 42,721 firm-year observations and 10,003 firms

We begin our sample generation process with 90,162 firm-year observations with available unconsolidated financial reports. We then drop observations for which we cannot calculate the firm-specific net tax benefit of debt due to missing observations for the dividend payout ratio. In addition, we drop firms with incomplete or non-existent ownership information and firms with negative debt ratios.^4^ Our final sample consists of 42,721 firm-year observations and 10,003 private firms.

We measure the debt ratio of our sample firms using the book debt to total assets ratio. Book debt is calculated as the sum of current and non-current liabilities.^5^ When accounting for firm-specific dividend payout policy, we include *d*, the firm-specific dividend payout ratio, into the calculation of the net tax benefit of debt. As we have only private firms in the sample, we cannot directly observe dividend payments. Instead, we calculate the firm-specific dividend payout ratio as a function of the firm’s profit/loss per period, *Profit*, and total shareholder funds, *SF*, as follows:

5 $$ d_{i,t} = \left\{ {\begin{array}{*{20}l} 0 \hfill & {\quad {\text{if}}\;{Profit}_{i,t} \le 0} \hfill \\ 1 \hfill & {\quad {\text{if}}\; {Profit}_{i,t} > 0 \;{\text{and}}\; {SF}_{i,t} - {SF}_{i,t - 1} \le 0} \hfill \\ 0 \hfill & {\quad {\text{if}}\;0 < {Profit}_{i,t} \le {SF}_{i,t} - {SF}_{i,t - 1} } \hfill \\ {\frac{{{\text{Profit}}_{i,t} - \left( {{\text{SF}}_{i,t} - {\text{SF}}_{i,t - 1} } \right)}}{{{\text{Profit}}_{i,t} }}} \hfill & {\quad {\text{otherwise}}} \hfill \\ \end{array} } \right. $$

In Eq. (5), *Profit* is the firm’s profit after taxes (net income) and *SF* is the firm’s total shareholder funds (total equity), calculated as total assets less current and non-current liabilities. Values for *d* vary between 0 (no profits distributed as dividends, all profits retained) and 1 (all profits distributed as dividends, no profits retained). If the increase in shareholder funds from *t* − 1 to *t* is larger than the observed profit in *t* we assume that all profits have been retained. In this case, the observed additional increase in shareholder funds must be due to a change in reserves that cannot be observed separately in the database.^6^

Using the firm-specific dividend payout ratio and tax rates from Table 1, we can compute the firm-specific net tax benefit of debt, NTBD_Payout_, as defined in Eq. (2). In Table 3, we present firm-specific average debt ratios, dividend payout ratios and net tax benefits of debt for all sample firms by year.

**Table 3:** Average firm-specific debt ratio, payout ratio and net tax benefit of debt by year

Years	Debt ratio		Payout ratio		NTBD_Payout_		*N*
	Mean	SD	Mean	SD	Mean	SD	
2002	0.6223	0.2442	0.1931	0.3331	0.2104	0.0972	629
2003	0.6231	0.2502	0.3699	0.4219	0.2211	0.0877	775
2004	0.6328	0.2543	0.4211	0.4168	0.1928	0.1069	1765
2005	0.6278	0.2483	0.2153	0.3505	0.1226	0.0835	2246
2006	0.6358	0.2681	0.1904	0.3403	0.1077	0.0737	2561
2007	0.7318	0.2894	0.2471	0.3551	0.1324	0.0656	4920
2008	0.7475	0.2974	0.2695	0.3641	0.1388	0.0631	4583
2009	0.7175	0.3315	0.4317	0.4252	0.1295	0.0459	7559
2010	0.6180	0.2994	0.4036	0.4239	0.1259	0.0490	5966
2011	0.6015	0.2905	0.2487	0.3707	0.1042	0.0614	5628
2012	0.6525	0.3505	0.3956	0.4268	0.1175	0.0653	6089
Total	0.6699	0.3071	0.3253	0.4023	0.1294	0.0694	42,721

This table shows the average firm-specific debt ratio (sum of current and non-current liabilities divided by total assets) as well as the average firm-specific payout ratio calculated as shown in Eq. (5). In addition, we present the average firm-specific net tax benefit of debt, calculated according to Gordon and MacKie-Mason (1990). For the calculation we use data from our sample consisting of 42,721 firm-year observations and 10,003 firms

The average debt ratio increases until 2008 and then decreases over the years 2009–2011. During the final year of our observation period, the average debt ratio increases slightly again. 2011 is the year with the lowest average debt ratio (61.80%) and 2008 is the year with the highest average debt ratio (74.75%). The average firm-specific dividend payout ratio of 32.53% shows that our sample firms on average distribute a third of their profits as dividends. In using only dividend tax rates to account for investor taxes we would thus not cover most of the investor level tax burden. Dividend payout ratios also vary over time. From 2005 to 2008, we observe dividend payout ratios below the sample mean, whereas dividend payout ratios in 2009, 2010 and 2012 are found to be above the sample mean.^7^ On average debt financing is preferred over equity financing and the average firm-specific net tax benefit of debt amounts to 12.94%. The advantage of debt financing has decreased over the years, being highest in the first 3 years of our sample period (2002–2004). In Table 4, we show average firm-specific debt ratios, dividend payout ratios and net tax benefits of debt for all sample firms by country.

**Table 4:** Average firm-specific debt ratio, payout ratio and net tax benefit of debt by country

Country	Debt ratio		Payout ratio		NTBD_Payout_		*N*
	Mean	SD	Mean	SD	Mean	SD	
Bulgaria	0.6419	0.3996	0.2319	0.3628	0.1088	0.0541	2322
Croatia	0.6269	0.5593	0.3681	0.4246	0.2131	0.0328	4104
Estonia	0.4331	0.2940	0.1885	0.3197	− 0.0886	0.0352	1266
Hungary	0.5682	0.2157	0.2774	0.3615	0.2745	0.1081	733
Latvia	0.7001	0.3676	0.3414	0.3874	0.0192	0.0754	618
Lithuania	0.5577	0.2229	0.2416	0.3572	0.0887	0.0351	1323
Poland	0.5518	0.3938	0.3095	0.4078	0.1051	0.0376	5151
Romania	0.6204	0.3042	0.3411	0.4111	0.1168	0.0621	4716
Russia	0.7475	0.5261	0.3531	0.4059	0.1302	0.0323	19,862
Slovakia	0.5202	0.2447	0.4131	0.4392	0.0451	0.0338	232
Ukraine	0.7445	0.4065	0.2754	0.3974	0.1937	0.0327	2394
Total	0.6699	0.3071	0.3253	0.4023	0.1295	0.0694	42,721

This table shows the average firm-specific debt ratio (sum of current and non-current liabilities divided by total assets) as well as the average firm-specific payout ratio calculated as shown in Eq. (5). In addition, we present the average firm-specific net tax benefit of debt, calculated according to Gordon and MacKie-Mason (1990). All values presented are country averages. For the calculation we use data from our sample consisting of 42,721 firm-year observations and 10,003 firms

Russian firms have the highest average debt ratio of 74.75%, closely followed by the Ukraine (74.45%). We observe the lowest debt ratios in Estonia (43.97%) and Slovakia (52.02%). The average debt ratio of Estonian firms is only 58.82% of the debt ratio of Russian firms, showing a large variation in average debt ratios among countries. This also holds for the average firm-specific dividend payout ratio. The highest average dividend payout ratio, found in Slovakia (41.31%), is more than twice the value of the lowest dividend payout ratio in Estonia (18.85%). Estonia is the only country with a negative average firm-specific net tax benefit of debt. The average net tax benefit of debt in Latvia and Slovakia is also close to zero, which means that in these countries tax treatment of debt financing is nearly as preferential as equity financing when considering firm-specific dividend payout policy and investor level taxation.

We integrate firm-level heterogeneity with respect to ownership into the analysis of capital structure choice. With the ownership data available, we can identify the type (individual, corporate, financial, fund, state), the nationality and percentage of direct ownership for all owners of the firm. Our analysis focuses on firms that have a domestic individual investor as the marginal owner. Contrary to Babbel et al. (2018), we do not consider foreign individual owners or non-individual owners as marginal owners in our analysis.

By comparing the percentage of ownership of all individual owners of a firm, we are able to identify the largest individual owner of the firm. For the empirical analysis, we divide firms into three groups based upon the percentage of the largest individual owner: (1) firms that are wholly owned by the largest individual owner (*WhollyOwn*), (2) firms for which the largest individual owner holds more than 50% of the shares and is thus the majority owner (*MajOwn*), and (3) firms for which the largest individual owner is the largest owner of the firm (*LargeOwn*). When defining our groups in this way, *MajOwn* and *LargeOwn* will automatically be equal to 1, if *WhollyOwn *= 1, but not vice versa.^8^ We therefore expect a larger number of firms to have an individual owner as the largest owner than to have an individual owner as the majority owner and a smaller number of firms to be fully owned by the largest individual owner. We use *WhollyOwn,* as Jiang et al. (2017) have shown that firms that are controlled by one single owner have the lowest agency costs and thus the most effective ownership structure. Alternatively, we follow Fossen and Simmler (2016) and use *MajOwn* to control for the effect of taxes in the presence of a majority owner and we follow Pindado and de la Torre (2011) and Babbel et al. (2018) who define the marginal owner of the firm as the largest owner *LargeOwn*.

In Table 5, we present the average combined holding of all individual owners, the average holding of the largest individual owner as well as the percentage of firms with an individual owner as the largest (majority) owner and the percentage of firms wholly owned by an individual.

**Table 5:** Statistics on individual owners by country, 2002–2012

Country	(1)	(2)	(3)	(4)	(5)
	Indiv. agg. (%)	Largest indiv. (%)	% firms large (%)	% firms major (%)	% firms wholly (%)
Bulgaria	78.94	56.44	77.85	48.09	19.92
Estonia	83.17	63.28	80.64	53.85	34.68
Croatia	78.50	68.26	77.52	64.51	50.45
Hungary	82.85	52.89	80.55	44.55	0.18
Lithuania	83.56	58.27	83.66	52.12	19.08
Latvia	90.06	65.91	87.87	63.99	31.16
Poland	87.83	59.43	90.08	47.66	15.52
Romania	79.51	60.75	78.25	54.21	22.88
Russia	83.54	77.01	83.48	74.88	58.81
Slovakia	90.72	82.21	98.77	83.95	61.73
Ukraine	78.72	49.51	77.19	41.05	6.57
Total	83.40	63.09	83.26	57.17	29.18

Column (1) in this table shows the average combined holding of individual owners per country and column (2) the average holding of the largest individual owners per country. Columns (3) to (5) show the percentage of firms for which (3) the largest owner is an individual owner, (4) the majority owner is an individual owner and (5) the percentage of firms wholly owned held by an individual. For the calculation we use data from our sample consisting of 42,721 firm-year observations and 10,003 firms

We can show that individual owners are, on average, the largest owner group for all countries in our sample as aggregated individual ownership is above 50% for all countries. Croatia (78.50%) and Ukraine (78.72%) are the countries with the lowest combined holding of individual owners. We find high values of combined individual ownership in Latvia (90.06%) and Slovakia (90.72%). In all countries except Ukraine, we find the largest individual owner, on average, to hold more than 50% of the shares of the firm. In Slovakia, the largest individual owner on average holds 82.21% of the firm, in Russia this value accounts for 77.01%. In 29.18% of all firm-years in our sample, the firm is wholly owned by an individual (*WhollyOwn* = 1), but values vary widely among our sample firms. Whereas 61.73% of Slovakian firms and 58.81% of Russian firms have one individual owner holding 100% of the firm, only 6.57% of firms from Ukraine and nearly no Hungarian firms (0.18%) are wholly owned by an individual. Ukraine and Hungary also show the lowest average percentage of firms for which the largest individual owner is the majority owner of the firm (41.05% and 44.55%, respectively). However, we find the majority of firms to have an individual owner as the majority owner. For all sample countries, more than 75% of the firms have an individual owner as the largest owner (*LargeOwn* = 1), the value again being highest for Slovakia (98.77%) followed by Poland (90.08%).

### Regression model

We are interested in the combined effects of investor taxation and firm heterogeneity on the capital structure choice of firms. Our dependent variable *DebtRatio* is defined by the book debt to total assets ratio. In our regressions, we use the following tax as well as firm- and country-level non-tax control variables.

Our main tax variable of interest is the firm-specific net tax benefit of debt. We address the potential endogeneity bias of dividend payout ratios in several ways. Firstly, we refrain from using the firm-specific net tax benefit of debt, but separately include the single tax rates that are used to calculate the net tax benefit of debt. Our first regression model therefore reads as follows:

6 $${Debt \,ratio_{i,t}}= \alpha + \beta_{1} \cdot \tau_{c} + \beta_{2} \cdot \tau_{i} + \beta_{3} \cdot \tau_{d} + \beta_{4} \cdot \tau_{g} + \beta_{{}} \cdot X_{i,t} + \beta_{{}} \cdot Y_{t} + u_{i} + v_{t} + \varepsilon_{i,t} $$

Secondly, we use two different specifications of the net tax benefit of debt. We start with the net tax benefit of debt as defined in Eq. (1), calculated based on the assumption that all corporate profits are distributed as dividends and thus *d *= 1. Next, we use the firm-specific dividend payout ratio to calculate NTBD_Payout_. We follow Graham (1999) in mitigating endogeneity concerns by using a 1-year lagged firm-specific dividend payout ratio. The second regression model reads as follows:

7 $${Debt \,ratio_{i,t}}= \alpha + \beta_{1} \cdot {\text{NTBD}}_{i,t} + \beta_{{}} \cdot X_{i,t} + \beta_{{}} \cdot Y_{t} + u_{i} + v_{t} + \varepsilon_{i,t} $$

Thirdly, we use six alternative definitions of the firm-specific dividend payout policy in the robustness checks in Sect. 5.

In all regression models, we include the same set of non-tax firm-level control variables $X_{i,t}$ and non-tax country-level control variables *Y*_*t*_. We follow the reasoning of Faccio and Xu (2015) and include firm-fixed effects $u_{i}$ and time-fixed effects $v_{t}$ to provide time-series evidence on the effect of changes in taxation on debt levels.

DeAngelo and Masulis (1980) show that debt ratios of firms are influenced by the existence of tax shields other than interest payments such as depreciation, investment tax credits or loss-carryforwards. Among the non-tax control variables, we implement *NOL* to control for other possibilities that generate tax shields (substitution hypothesis). It is a dummy variable taking the value 1 if there is a negative EBIT in the previous year and 0 otherwise. We expect a negative coefficient for *NOL*.

Previous studies such as Wald (1999) found that the profitability of a firm has an influence on its debt ratio. There are several theories regarding in which direction profitability influences the debt ratio. According to the trade-off theory more profitable firms should have higher debt ratios as there is a lower risk of financial distress, see Kraus and Litzenberger (1973). The free cash flow theory also suggests that more profitable firms will have higher debt ratios (see Jensen 1986), while the pecking order theory argues that firms with investment opportunities are more profitable and less levered, see Myers and Majluf (1984). We calculate *Profitability* as the EBIT deflated by total assets, both lagged by 1 year. To control for size effects, we add *Size* to our model, measured as the natural logarithm of total assets as suggested by Schulman et al. (1996). Larger corporations are found to have higher debt ratios, which is why we expect a positive coefficient for *Size*. Additionally, we include *Tangibles*, tangible assets deflated by total assets, into our regression model. Again, previous literature has found ambiguous effects of tangibles on debt financing. On the one hand, the costs of financial distress are expected to be lower, the higher the tangible assets are as they serve as collateral (see Scott 1977; Harris and Raviv 1990). On the other hand, following the discussion of DeAngelo and Masulis (1980), higher tangible assets lead to higher non-debt tax shields related to tangible assets such as depreciation deductions or investment credits that crowd out the positive effect of interest deduction. Pfaffermayr et al. (2013) show that the debt ratio of a firm changes throughout its life cycle. We therefore add the variable *Age*, calculated as the natural logarithm of the years between incorporation and the year under investigation, to test whether older firms have smaller debt ratios and we expect a negative coefficient for *Age*. As we measure the debt ratio based on unconsolidated accounts, it also includes intra-group debt. Huizinga et al. (2008) as well as Büttner et al. (2012) document the importance of internal debt financing for corporate groups. We control for the possibility to have access to intra-group debt by using the variable *Standalone.* It takes the value 1 if the firm either has at least one majority-owned subsidiary or if it is majority-owned by another corporation.

To control for time-variant country-specific effects, we add several country-level variables to the model. An important aspect with respect to debt financing is creditor rights, i.e., law enforcement in the given country. We use the rule of law estimate of the World Bank, *Law*, to control for enforcement of creditor rights. During our observation period, most of the CEE countries analyzed became EU member states. EU membership offers new opportunities for international financing. *EU* is a dummy variable taking the value 1 if the country is an EU member in the current year and 0 otherwise. Ways of financing have been found to depend on the size of the country’s capital market. We control for this effect by integrating *Market*, calculated as stock market capitalization deflated by the GDP. Additionally, we control for the annual growth in GDP per capita, *GDPGrowth*, as well as the annual percentage change in consumer prices, *Inflation*, both again obtained from the World Bank. To reduce the effect of outliers, we winsorize all continuous control variables at the 1st and 99th percentiles.

To test Hypotheses 2a and 2b, we add information on firm-specific individual ownership to our regression models. We include three different measures of individual ownership as shown in Table 5. We expect the influence of the firm-specific net tax benefit of debt on the debt ratio to be higher if the marginal owner is an individual owner (Hypothesis 2a) and highest for firms that are wholly owned by one individual (Hypothesis 2b). To test these hypotheses, we separately interact the tax variables from the regression models shown in Eqs. (6) and (7) with the three dummy variables *WhollyOwn*, *MajOwn* and *LargeOwn* to control for different levels of individual ownership.

Table 6 shows summary statistics for all control variables included in our regression analysis.

**Table 6:** Summary statistics, 2002–2012

Variable	Obs	Mean	Std.Dev.	Min	Max
$\tau_{c}$	42,721	0.1928	0.0474	0	0.35
$\tau_{i}$	42,721	0.1249	0.0604	0	0.26
$\tau_{d}$	42,721	0.1089	0.0552	0	0.26
$\tau_{g}$	42,721	0.1194	0.0801	0	0.29
NTBD	42,721	0.1566	0.0617	− 0.10	0.37
NTBD_Payout_	42,721	0.1294	0.0682	− 0.13	0.37
NOL	42,721	0.1665	0.3725	0	1
Profitability	42,721	0.0518	0.1368	− 3.86	9.51
Size	42,721	16.7692	1.0169	6.91	24.85
Tangibles	42,721	0.2978	0.2700	0	1
Age	42,721	31.2121	36.7973	0	301
Standalone	42,721	0.6210	0.4851	0	1
Law	42,721	− 0.3219	0.6300	− 0.99	1.17
EU	42,721	0.2880	0.4528	0	1
Market	42,721	0.4413	0.2956	0.04	1.16
GDPGrowth	42,721	2.8852	5.6804	− 14.56	12.92
Inflation	42,721	7.3329	4.1233	− 1.07	25.23

This table shows summary statistics for the tax rates $(\tau_{c} , \tau_{i} , \tau_{d}  \;{\text{and}}\; \tau_{g} )$ and the two different specifications of the net tax benefit of debt (NTBD and NTBD_Payout_) used in the regression analyses. Additionally, summary statistics for the non-tax firm-level and country-level control variables are shown. Firm-level control variables include *NOL*, a dummy variable taking the value 1 if there is a negative EBIT in the year before, *Profitability* (EBIT deflated by total assets), *Size* (natural log of total assets), *Tangibles* (tangible assets deflated by total assets), *Age* (firm age in years) and *Standalone* (dummy variable if the firm does not belong to a corporate group). Country-level control variables are *Law* (rule of law estimate of the WorldBank), *EU*, a dummy variable taking the value 1 if the country is an EU member in the current year, *Market* (stock market capitalization deflated by GDP), *GDPGrowth* (annual growth of GDP per capita) and *Inflation* (annual change in consumer prices). Statistics are calculated based on 42,721 firm-year observations from 10,003 firms

Controlling for the substitution hypothesis, we identify only 16.65% of our observations to have a tax-loss-carryforward. Although we observe a low number of loss firms, the average profitability is also low, accounting for only 5.18%. The oldest firm in the sample is 301 years old, but the average value (31.21 years) is far lower. 62.10% of all firms in the sample are standalone firms, which means that they neither have majority-owned subsidiaries nor are majority-owned by other corporations.

## The effect of individual taxes and firm heterogeneity on capital structure choice

### Effects of single tax rates

We start our empirical analysis by estimating separate effects for the tax rates on corporate income ($\tau_{c}$) as well as tax rates on investor level interest income ($\tau_{i}$), dividend income ($\tau_{d}$) and capital gains ($\tau_{g}$). According to our theoretical predictions, we expect a negative coefficient for $\tau_{i}$ and a positive coefficient for all other tax rates in the sample.^9^ We present the regression results in column (1) in Table 7.

**Table 7:** Investor level tax rates, ownership and capital structure choice, 2002–2012

Variables	Specification				
	(1)	(1a)	(2)	(3)	(4)
	Baseline	Baseline d*y*/d*x*	*OWN *=* LargeOwn*	*OWN *=* MajOwn*	*OWN *=* WhollyOwn*
$\tau_{c}$	0.4111*** (0.0888)	0.1252	0.5097*** (0.1289)	0.5114*** (0.1313)	0.4312*** (0.0904)
$\tau_{i}$	− 0.1816*** (0.0493)	− 0.0339	0.1339 (0.0843)	0.1294 (0.0860)	0.1413 (0.1153)
$\tau_{d}$	0.3851*** (0.0441)	0.0624	0.0512 (0.0610)	0.0530 (0.0619)	0.4271*** (0.0475)
$\tau_{g}$	0.0958*** (0.0367)	0.0185	0.0736 (0.0616)	0.0712 (0.0625)	0.0521 (0.0400)
OWN			0.0397 (0.0259)	0.0317 (0.0267)	− 0.0394 (0.0273)
$\tau_{c}$·OWN			− 0.1090 (0.1059)	− 0.1048 (0.1095)	0.1572 (0.0998)
$\tau_{i}$·OWN			− 0.3811*** (0.0821)	− 0.3708*** (0.0837)	− 0.2108*** (0.0511)
$\tau_{d}$·OWN			0.1966** (0.0984)	0.2243** (0.1003)	0.2840*** (0.1051)
$\tau_{g}$·OWN			0.2068** (0.0953)	0.1824* (0.0972)	0.1567** (0.0761)
NOL	0.0560*** (0.0040)	0.0142	0.0561*** (0.0040)	0.0561*** (0.0040)	0.0561*** (0.0040)
Profitability	− 0.0067*** (0.0021)	− 0.0006	− 0.0067*** (0.0021)	− 0.0067*** (0.0021)	− 0.0067*** (0.0021)
Size	− 0.0122*** (0.0020)	− 0.2017	− 0.0122*** (0.0020)	− 0.0122*** (0.0020)	− 0.0120*** (0.0020)
Tangibles	− 0.0730*** (0.0100)	− 0.0353	− 0.0720*** (0.0100)	− 0.0720*** (0.0100)	− 0.0751*** (0.0100)
Age	0.0005*** (0.0001)	0.0267	0.0005*** (0.0001)	0.0005*** (0.0001)	0.0005*** (0.0001)
Standalone	0.1154*** (0.0213)	− 0.0155	0.1191*** (0.0213)	0.1182*** (0.0213)	0.1121*** (0.0213)
Law	− 0.1260*** (0.0239)	0.0639	− 0.1289*** (0.0240)	− 0.1289*** (0.0240)	− 0.0816*** (0.0269)
EU	− 0.0581*** (0.0067)	− 0.0234	− 0.0563*** (0.0068)	− 0.0557*** (0.0068)	− 0.0603*** (0.0069)
Market	0.0206** (0.0102)	0.0141	0.0220** (0.0102)	0.0218** (0.0102)	0.0139 (0.0103)
GDPGrowth	− 0.0022*** (0.0007)	− 0.0146	− 0.0022*** (0.0007)	− 0.0022*** (0.0007)	− 0.0022*** (0.0007)
Inflation	0.0004 (0.0006)	0.0045	0.0004 (0.0006)	0.0004 (0.0006)	0.0004 (0.0006)
Firm-FE	Yes		Yes	Yes	Yes
Year-FE	Yes		Yes	Yes	Yes
*N*	42,721		42,721	42,721	42,721
*R* ^2^	0.5687		0.5688	0.5688	0.5688

This table shows regression results for Eq. (6), investigating the influence of investor level tax rates and firm-specific ownership on capital structure choice. In all regressions, firm- and year-fixed effects are included. Robust standard errors, clustered at country-level, are presented in parentheses

***, **, and * indicate statistical significance at the 1%, 5%, and 10% levels, respectively

Our results support the expected positive effect of the corporate tax rate as well as the dividend tax rate and the capital gains tax rate and the expected negative effect of the interest tax rate on debt levels. An increase in the corporate tax rate (dividend tax rate) by 10 percentage points increases debt ratios by about 4.1 (3.8) percentage points, holding other tax rates constant. Whereas the results for the corporate tax rate are in line with the results of Faccio and Xu (2015), we find larger effects for the dividend tax rate. Among our firm-level control variables, we find significant positive effects for *NOL* and *Age* and significant negative effects for *Size, Profitability* and *Tangibles*. According to the substitution hypothesis, loss-carryforwards serve as an additional tax shield that lowers the tax effect of interest deductibility, which is why we would expect a negative coefficient for *NOL*. However, the value of other tax shields also depends on the corporate tax rate in the country. We therefore multiply *NOL* by the statutory corporate tax rate, $\tau_{c}$ and include the interaction term in our regression analysis. Non-tabulated results show that the coefficient for the interaction term (significant at the 5% level) is now negative and accounts for − 0.1488, in line with our expectation.

In column (1a), we assess the economic significance of the baseline results. We follow Faccio and Xu (2015) and present an elasticity measure (d*y*/d*x*), computed at mean values of *x* and *y*. The values in column (1a) therefore show the percentage increase in debt ratios due to a 1% increase in the respective independent variables. Results for the elasticities show that taxes are an important determinant of capital structure choice. In line with the results of Faccio and Xu (2015), we find that corporate taxes have the highest importance. A 1% increase in the corporate tax rate leads to an increase in the debt ratio of 0.1252%. Among the personal tax rates, dividend taxes have the most important influence on the debt ratio. A 1% increase in the dividend tax rate leads to an increase in the debt ratio of 0.0624%, whereas a 1% increase in the capital gains tax rate increases leverage by only 0.0185%. The elasticity of our firm-specific and country-specific control variables in general are smaller than those of the corporate and dividend tax rate. The only firm-specific control variable that is economically more important than taxes is firm size. Again, this finding is in line with Faccio and Xu (2015). Among the country-specific control variables, we find the rule of law estimate to also have a notable economic importance for debt levels.

In columns (2)–(4) in Table 7, we add information on firm-specific ownership to the regression analysis. We interact the tax rates from specification (1) with three different ownership dummies. When adding the interaction terms to the model, we expect a significant baseline effect only for the corporate tax rate as it is the only non-investor level tax rate in the model. Furthermore, we expect significant positive coefficients for the interaction of the ownership dummies with the dividend and capital gains tax rate and a significant negative coefficient for the interaction of the ownership dummies with the interest tax rate. Results in columns (2)–(4) support our expectations. The coefficient for $\tau_{c}$ is positive and significant in all specifications, whereas the interaction of the corporate tax rate and the ownership dummies shows no significant results. Contrary to this, we do not find significant baseline effects for $\tau_{i}$, $\tau_{d}$ and $\tau_{g}$, but significant coefficients for the interaction terms with our ownership dummies. The only exception to this is the baseline coefficient for $\tau_{d}$, which is positive and significant in column (4). However, in specification (4), the interaction term refers to firms that are wholly owned by an individual; thus, the baseline effect also captures the effect for firms having an individual owner as the largest owner or as the majority owner.

Comparing the results from columns (2)–(4), we see that the effect of the dividend tax rate on debt ratios increases in the level of individual ownership. In column (4), the combined effect of the dividend tax rate on debt ratios for firms that are wholly owned by an individual is 0.7111 (= 0.4271 + 0.2840). This is nearly three times the effect for firms in which only the largest owner is an individual owner (0.2478 = 0.0512 + 0.1966). The effect of the capital gains tax rate on debt ratios, however, decreases with the level of individual ownership. The overall effect of capital gains taxes in column (2), referring to firms with an individual owner as the largest owner, is 0.2804 (= 0.0736 + 0.2068) and decreases to 0.2088 (= 0.0521 + 0.1567) in column (4) for firms wholly owned by an individual. We attribute the decreasing effect of capital gains taxes to liquidity issues. The smaller the number of shares held by individual owners, the more likely it is that they can be sold.

Even for firms with the lowest combined effect of capital gains taxes in column (4), the combined effect of capital gains taxes on debt ratios is nearly twice the effect found in the baseline specification in column (1), where we did not control for firm-specific ownership. This shows the importance of jointly analyzing capital gains taxes and firm-specific ownership.

In a next step, we include the firm-specific dividend payout ratio in our analysis. We follow Faccio and Xu (2015) and add the dummy variable *NoDivPayer* to the model. This dummy variable takes the value 1 if a firm does not pay any dividends over the whole observation period. We interact *NoDivPayer* with $\tau_{d}$ and expect a negative coefficient for the interaction term. 1027 (9.74%) of the 10,003 firms in our sample do not pay dividends over the whole observation period, which is somewhat lower than the 29% found by Faccio and Xu (2015). In Table 8, we show the results for the interacted regression model.

**Table 8:** Investor level tax rates, ownership, payout policy and capital structure choice, 2002–2012

Variables	Specification			
	(1)	(2)	(3)	(4)
	Baseline NoDivPayer	*OWN *=* LargeOwn* NoDivPayer	*OWN *=* MajOwn* NoDivPayer	*OWN *=* WhollyOwn* NoDivPayer
$\tau_{c}$	0.4212*** (0.0888)	0.5195*** (0.1290)	0.5163*** (0.1313)	0.4309*** (0.0904)
$\tau_{i}$	− 0.1811*** (0.0493)	0.1272 (0.0843)	0.1296 (0.0860)	0.1440 (0.1154)
$\tau_{d}$	0.3441*** (0.0449)	0.0603 (0.0610)	0.0555 (0.0619)	0.4263*** (0.0475)
$\tau_{g}$	0.0989*** (0.0367)	0.0671 (0.0616)	0.0674 (0.0625)	0.0522 (0.0400)
OWN		0.0359 (0.0259)	0.0324 (0.0267)	− 0.0402 (0.0273)
$\tau_{c}$·OWN		− 0.1058 (0.1059)	− 0.1098 (0.1095)	0.1613 (0.0999)
$\tau_{i}$·OWN		− 0.3630*** (0.0821)	− 0.3669*** (0.0837)	− 0.2108*** (0.0511)
$\tau_{d}$·OWN		0.1998** (0.0984)	0.2320** (0.1003)	0.2810*** (0.1051)
$\tau_{g}$·OWN		2114** (0.0953)	0.1815* (0.0972)	0.1576** (0.0761)
$\tau_{d} \cdot {\text{NoDivPayer}}$	− 0.1030*** (0.0221)			
$\tau_{d}$·OWN·${\text{NoDivPayer}}$		− 0.0882*** (0.0276)	− 0.0897*** (0.0280)	− 0.0396 (0.0309)
Firm-controls	Yes	Yes	Yes	Yes
Country-controls	Yes	Yes	Yes	Yes
Firm-FE	Yes	Yes	Yes	Yes
Year-FE	Yes	Yes	Yes	Yes
*N*	42,721	42,721	42,721	42,721
*R* ^2^	0.5688	0.5687	0.5689	0.5688

This table shows regression results for Eq. (6), investigating the influence of investor level tax rates, firm-specific ownership and firm-specific dividend payout policy on capital structure choice. In all regressions, firm-level as well as country-level control variables from Table 7 as well as firm- and year-fixed effects are included, but not reported. Robust standard errors, clustered at country-level, are presented in parentheses

***, **, and * indicate statistical significance at the 1%, 5%, and 10% levels, respectively

Consistent with our prediction, we find a significantly negative coefficient for interaction of the dividend tax rate and firms not paying dividends in column (1). In columns (2)–(4), we test the joint effect of firm-specific dividend payout ratios and firm-specific ownership. In columns (2) and (3), we find the expected negative coefficient for the triple interaction term for firms with an individual as the largest owner or the majority owner. In column (4), we find a negative coefficient, that is not, however, significant at conventional levels for firms that are wholly owned by an individual. As already shown in Table 7, the baseline coefficient for $\tau_{d}$ is again statistically significant and potentially influencing the statistical significance of the interaction term. The significant results for most of the specifications make us confident that dividend taxes are of less importance for a firm’s debt ratio if the firm does not pay dividends.^10^ This is first evidence that it is important to jointly analyze firms-specific dividend payout policy and ownership in capital structure research.

### Effects of the net tax benefit of debt

In this section, we present regression results for the estimation of the effect of the net tax benefit of debt rather than single tax rates. In the first analysis, we use the net tax benefit of debt as shown in Eq. (1) and thus assume that all profits are distributed to owners as dividends (i.e., *d *= 1). We present the results in Table 9.

**Table 9:** Investor net tax benefit of debt, ownership and capital structure choice, 2002–2012

Variables	Specification			
	(1)	(2)	(3)	(4)
	Baseline	*OWN *=* LargeOwn*	*OWN *=* MajOwn*	*OWN *=* WhollyOwn*
NTBD	0.2680*** (0.0351)	− 0.0461 (0.0931)	− 0.0326 (0.0627)	0.0625 (0.0557)
OWN		− 0.0307* (0.0182)	− 0.0527*** (0.0163)	− 0.0286 (0.0176)
NTBD·OWN		0.3083*** (0.1089)	0.3380*** (0.0986)	0.3503*** (0.1011)
Firm-controls	Yes	Yes	Yes	Yes
Country-controls	Yes	Yes	Yes	Yes
Firm-FE	Yes	Yes	Yes	Yes
Year-FE	Yes	Yes	Yes	Yes
*N*	42,721	42,721	42,721	42,721
*R* ^2^	0.5652	0.7247	0.7248	0.7248
Combined effect (= NTBD + NTBD·OWN)	0.2680	0.2622	0.3054	0.4128

This table shows regression results for Eq. (7), investigating the influence of the investor level net tax benefit of debt and firm-specific ownership on capital structure choice. In all regressions, firm-level as well as country-level control variables from Table 7 as well as firm- and year-fixed effects are included, but not reported. Robust standard errors, clustered at country-level, are presented in parentheses

***, **, and * indicate statistical significance at the 1%, 5%, and 10% levels, respectively

Column (1) in Table 9 gives the results that consider investor level taxation, but ignores firm-specific dividend payout policy and ownership. In line with hypothesis 1, we obtain a significant positive coefficient for the net tax benefit of debt of 0.2680. An increase in the net tax benefit of debt by 10 percentage points leads to an increase in the debt ratio of about 2.68 percentage points.^11^ As in Table 8, we calculate elasticities for all coefficients in column (1). Non-tabulated results show that an increase in the net tax benefit of debt of 1% results in an increase in debt ratios of 0.0668%. As in Table 8, we find only firm size and the rule of law estimator to have a similar economic importance on debt levels.

In columns (2)–(4), we add firm-specific ownership to the regression analysis, but do not consider firm-specific dividend payout policy. The coefficient of the interaction term NTBD·OWN is significant and has the expected positive sign for all three definitions of the marginal owners. The combined effect of the net tax benefit of debt on debt ratios in column (2) is 0.2622 if the largest owner is an individual owner and rises to 0.3054 in column (3) if the majority owner is an individual owner. It is 0.4128 and is thus highest in column (4) if the firm is wholly owned by one individual. Consistent with Hypothesis 2a, we find that the effect of the net tax benefit of debt on debt ratios is higher if the marginal owner is a domestic individual investor. The effect for firms wholly owned by an individual is about 55% higher than the effect for firms with an individual as the largest owner, which supports Hypothesis 2b. Controlling for firm-specific ownership has a substantial impact on the effect of the net tax benefit of debt on capital structure choice, especially if firms are either held by a majority individual owner or wholly owned by an individual. For firms that have an ownership structure with low agency costs, the effect of the net tax benefit of debt on debt ratios is about 1.55 times higher than for firms that have an ownership structure with high agency costs.

In our last analysis, we use the firm-specific dividend payout ratio to calculate NTBD_Payout_ as shown in Eq. (2). In Table 10, we present the results for the effect of NTBD_Payout_ on debt ratios.

**Table 10:** Investor net tax benefit of debt, ownership, payout policy and capital structure choice, 2002–2012

Variables	Specification			
	(1)	(2)	(3)	(4)
	Baseline	*OWN *=* LargeOwn*	*OWN *=* MajOwn*	*OWN *=* WhollyOwn*
NTBD_Payout_	0.0806** (0.0315)	− 0.0839 (0.0842)	− 0.0203 (0.0695)	0.0527 (0.0534)
OWN		− 0.0269* (0.0147)	− 0.0495*** (0.0135)	− 0.0377*** (0.0145)
NTBD_Payout_·OWN		0.3445*** (0.0968)	0.3903*** (0.0832)	0.4863*** (0.0885)
Firm-controls	Yes	Yes	Yes	Yes
Country-controls	Yes	Yes	Yes	Yes
Firm-FE	Yes	Yes	Yes	Yes
Year-FE	Yes	Yes	Yes	Yes
*N*	42,721	42,721	42,721	42,721
*R* ^2^	0.5648	0.7247	0.7248	0.7248
Combined effect (= NTBD_Payout_ + NTBD_Payout_·OWN)	0.0806	0.2606	0.3700	0.5390

This table shows regression results for Eq. (6), investigating the influence of the investor-level net tax benefit of debt, firm-specific dividend payout policy and firm-specific ownership on capital structure choice. In all regressions, firm-level as well as country-level control variables from Table 7 as well as firm- and year-fixed effects are included, but not reported. Robust standard errors, clustered at country-level, are presented in parentheses

***, **, and * indicate statistical significance at the 1%, 5%, and 10% levels, respectively

We have shown in Tables 3 and 4 that only about 32% of all profits are distributed to shareholders as dividends, whereas the rest are retained and therefore subject to future capital gains taxation. Low firm-specific dividend payout ratios as well as effective capital gains tax rates that are lower than dividend tax rates^12^ both lead to a reduction in the tax rate on income from equity. As taxation of equity decreases, the net tax advantage of debt also decreases. Additionally, the mean value of NTBD_Payout_, as shown in Table 6, is 0.1294 and is lower than the mean value of NTBD, which is 0.1566. However, there is a very high correlation between our two measures of the net tax benefit of debt, as reflected by a Pearson’s correlation coefficient of 0.8672.

We expect NTBD_Payout_ to be a more precise measure of the effect of investor level taxes on debt ratios compared to NTBD as it includes firm-specific dividend payout ratios and capital gains taxes. For our expectation to hold, coefficients in Table 10 must be higher than in Table 9 due to less measurement error. However, the coefficient for NTBD_Payout_ in the baseline specification in column (1) in Table 10 is 0.0806 and is thus lower than the coefficient for NTBD in column (1) in Table 9 (0.2680). In the baseline specification, we do not control for firm-specific ownership; thus, the expected positive effect of a more precise measurement of the net tax benefit of debt by including firm-specific dividend payout policy is outweighed by the measurement error that occurs when ignoring firm-specific ownership. Using the firm-specific dividend payout ratio reflects a more accurate calculation of the investor level net tax benefit of debt, as both dividend and capital gains tax rates are included. However, for this additional information to reduce overall measurement error, it is crucial that the investor level tax rates used in the calculation of NTBD_Payout_ truly represent the tax rates of the marginal investor. If this is not the case, adding capital gains taxes to the model increases the measurement error and thus the downward bias on the coefficient.

As soon as we control for firm-specific ownership, as shown in columns (2) to (4), we find all combined effects of NTBD_Payout_ to be larger than those in Table 9, consistent with our prediction of a more precise measurement. In line with Hypotheses 2a and 2b, the effect is higher if the marginal investor is an individual and the effect increases in the level of individual ownership.

Next, we compare our results from Table 10 with the baseline result of the effect of the net tax benefit of debt on debt ratios as shown in column (1) in Table 9. Recall that the latter accounts for 0.2680 and does not take either firm-specific ownership nor firm-specific dividend payout policy into account. We find a similar combined effect of 0.2606 in column (2) in Table 10. For firms with an individual as the largest owner, adding information on firm-specific ownership and firm-specific dividend payout policy does not influence the effect of the net tax benefit of debt on debt ratios. In other words, the measurement error of not considering firm-specific ownership and firm-specific dividend payout policy is negligibly small for firms with an individual as the largest owner.

If, however, firms are majority-owned by an individual, controlling for firm-specific ownership and firm-specific dividend payout policy increases the effect of the net tax benefit of debt on debt ratios by 38% (0.3700 vs. 0.2680). The strongest increase can be found in column (4). If a firm is wholly owned by an individual, controlling for firm-specific ownership and firm-specific dividend payout policy more than doubles the effect of the net tax benefit of debt on debt ratios. Depending on the marginal owner of the firm, jointly controlling for firm-specific ownership and firm-specific dividend payout policy can substantially enhance the understanding of the effect of investor level taxes on debt ratios.

## Robustness tests

In Eq. (2), the investor level tax rate on equity is determined by both the taxation of dividends and capital gains. Whereas dividends are taxed upon distribution, capital gains are taxed at realization, offering owners the possibility to defer capital gains taxation. In our calculations of the net tax benefit of debt, we therefore use an effective capital gains tax rate rather than the statutory marginal tax rate on capital gains and multiply the statutory marginal tax rate on capital gains by *α* = 0.5. We test whether our results are influenced by the choice of *α* and re-calculate NTBD_Payout_, assuming that *α* = 0.25 as suggested by Feldstein and Summers (1979). Also, we ignore the benefit arising from the deferral of capital gains taxation and re-calculate NTBD_Payout_, assuming a full-scale effect on capital structure not only for dividend taxes, but also for taxes on capital gains, thus *α* = 1. Our results are presented in Table 11.

**Table 11:** Alternative Measures of the Effective Capital Gains Tax Rate, 2002–2012

Variables	Specification					
	(1a)	(1b)	(1c)	(2a)	(2b)	(2c)
	*OWN *=* LargeOwn*	*OWN *=* MajOwn*	*OWN *=* WhollyOwn*	*OWN *=* LargeOwn*	*OWN *=* MajOwn*	*OWN *=* WhollyOwn*
*α*	*α* = 0.25	*α* = 0.25	*α* = 0.25	*α* = 1	*α* = 1	*α* = 1
NTBD_Payout_	− 0.0742 (0.0695)	− 0.0465 (0.0580)	0.0063 (0.0465)	0.0311 (0.0820)	0.0373 (0.0811)	0.1246** (0.0575)
OWN	− 0.0149 (0.0119)	− 0.0359*** (0.0110)	− 0.0212* (0.0121)	− 0.0279 (0.0176)	− 0.0658*** (0.0182)	− 0.0629*** (0.0188)
NTBD_Payout_·OWN	0.2856*** (0.0718)	0.3257*** (0.0736)	0.4073*** (0.0790)	0.2834*** (0.0961)	0.4105*** (0.0907)	0.5524*** (0.0959)
Firm-controls	Yes	Yes	Yes	Yes	Yes	Yes
Country-controls	Yes	Yes	Yes	Yes	Yes	Yes
Firm-FE	Yes	Yes	Yes	Yes	Yes	Yes
Year-FE	Yes	Yes	Yes	Yes	Yes	Yes
*N*	42,721	42,721	42,721	42,721	42,721	42,721
*R* ^2^	0.7247	0.7248	0.7248	0.7247	0.7248	0.7248
Combined effect (= NTBD_Payout_ + NTBD_Payout_·OWN)	0.2114	0.2792	0.4136	0.3145	0.4478	0.6770

This table shows regression results for different measures of the capital gains discounting factor, *α*. In all regressions, firm-level as well as country-level control variables from Table 7 as well as firm- and year-fixed effects are included, but not reported. Robust standard errors, clustered at country-level, are presented in parentheses

***, **, and * indicate statistical significance at the 1%, 5%, and 10% levels, respectively

Considering different definitions of the capital gains tax discount factor *α* and different definitions of the marginal owner of the firm, we find results similar to our main findings in Table 10. If we lower *α* to 0.25, we still find a positive and significant combined effect of the net tax benefit of debt on debt ratios of 0.2114, if the largest owner of the firm is an individual owner. Again, the effect nearly doubles if all shares of the firm are wholly owned by an individual. If we assume that investors do not discount capital gains taxes (*α* = 1), the combined effect of the net tax benefit of debt on debt ratios increases to 0.3145, if the largest owner of the firm is an individual owner. This is higher than the coefficient found in specification (2) of Table 9 and an indicator that a value of *α* below 1 can be another potential source of measurement error in the calculation of the net tax benefit of debt.

Integrating the firm-specific dividend payout policy into the calculation of the net tax benefit of debt might cause endogeneity issues. In Table 10, we have followed the reasoning of Graham (1999) and have used the firm-specific dividend payout ratio lagged by 1 year for the calculation of the net tax benefit of debt. We now present six alternative measures for the firm-specific dividend payout ratio in order to see whether our main results still hold.

We use (1) a 3-year moving average of the firm-specific dividend payout ratio and (2) a country-year average dividend payout ratio. Also, we follow Faccio and Xu (2015) and use (3) a blended average of taxes on dividends and capital gains. Additionally, we stick to the firm-specific dividend payout ratio lagged by 1 year as in Table 10, but (4) use an alternative definition of shareholder funds that excludes subscribed capital. In another specification, we use (5) a firm-specific average dividend payout ratio, calculated as follows^13^:

8 $$ \frac{{\mathop \sum \nolimits_{t = 1}^{T} {Profit}_{i,t} - \left( {{{SF}}_{i,t} - {{SF}}_{i,t - 1} } \right)}}{{\mathop \sum \nolimits_{t = 1}^{T} {{Profit}}_{i,t} }}. $$

If we consider the sum of profits rather than single-year observations of a firm’s profit, we can calculate firm-average payout ratios including years with losses. Lastly, we assume (6) that the firm does not pay dividends at all and thus, *d *= 0 for all sample years.

In Table 12, we present results for NTBD_Payout_ and the interaction term NTBD_Payout_· OWN for the three different definitions of the marginal owner.

**Table 12:** Alternative measures of firm-specific dividend payout policy, 2002–2012

Variables	Specification					
	(1a)	(1b)	(1c)	(2a)	(2b)	(2c)
	*OWN *=* LargeOwn*	*OWN *=* MajOwn*	*OWN *=* WhollyOwn*	*OWN *=* LargeOwn*	*OWN *=* MajOwn*	*OWN *=* WhollyOwn*
*d*	3 years moving avg.	3 years moving avg.	3 years moving avg.	Country-year avg.	Country-year avg.	Country-year avg.
NTBD_Payout_	− 0.0523 (0.0935)	− 0.0041 (0.0792)	0.0857 (0.0653)	− 0.0537 (0.0947)	0.0259 (0.0684)	0.1100* (0.0582)
OWN	− 0.0291* (0.0162)	− 0.0566*** (0.0149)	− 0.0445*** (0.0158)	− 0.0323** (0.0159)	− 0.0540*** (0.0137)	− 0.0409*** (0.0153)
NTBD_Payout_·OWN	0.3701*** (0.1115)	0.4530*** (0.0991)	0.5326*** (0.1000)	0.3806*** (0.1090)	0.4197*** (0.0926)	0.5032*** (0.0971)
Firm-controls	Yes	Yes	Yes	Yes	Yes	Yes
Country-controls	Yes	Yes	Yes	Yes	Yes	Yes
Firm-FE	Yes	Yes	Yes	Yes	Yes	Yes
Year-FE	Yes	Yes	Yes	Yes	Yes	Yes
*N*	42,721	42,721	42,721	42,721	42,721	42,721
*R* ^2^	0.7274	0.7275	0.7274	0.7248	0.7247	0.7248
Combined effect (= NTBD_Payout_ + NTBD_Payout_·OWN)	0.3178	0.4489	0.6183	0.3269	0.4456	0.6132

Variables	Specification					
	(3a)	(3b)	(3c)	(4a)	(4b)	(4c)
	*OWN *=* LargeOwn*	*OWN *=* MajOwn*	*OWN *=* WhollyOwn*	*OWN *=* LargeOwn*	*OWN *=* MajOwn*	*OWN *=* WhollyOwn*
*d*	0.5	0.5	0.5	Only other SF	Only other SF	Only other SF
NTBD_Payout_	− 0.0503 (0.0963)	0.0216 (0.0698)	0.1141* (0.0585)	− 0.0061 (0.0868)	0.0606 (0.0642)	0.1414** (0.0642)
OWN	− 0.0332** (0.0166)	− 0.0564*** (0.0147)	− 0.0406** (0.0161)	− 0.0282* (0.0157)	− 0.0505*** (0.0139)	− 0.0358** (0.0150)
NTBD_Payout_·OWN	0.3724*** (0.1111)	0.4188*** (0.0972)	0.4831*** (0.1006)	0.3510*** (0.1067)	0.3914*** (0.1004)	0.4519*** (0.0963)
Firm-controls	Yes	Yes	Yes	Yes	Yes	Yes
Country-controls	Yes	Yes	Yes	Yes	Yes	Yes
Firm-FE	Yes	Yes	Yes	Yes	Yes	Yes
Year-FE	Yes	Yes	Yes	Yes	Yes	Yes
*N*	42,721	42,721	42,721	42,721	42,721	42,721
*R* ^2^	0.7248	0.7247	0.7248	0.7248	0.7247	0.7248
Combined effect (= NTBD_Payout_ + NTBD_Payout_·OWN)	0.3221	0.4404	0.5972	0.3449	0.4520	0.5933

Variables	Specification					
	(5a)	(5b)	(5c)	(6a)	(6b)	(6c)
	*OWN *=* LargeOwn*	*OWN *=* MajOwn*	*OWN *=* WhollyOwn*	*OWN *=* LargeOwn*	*OWN *=* MajOwn*	*OWN *=* WhollyOwn*
*d*	Firm avg.	Firm avg.	Firm avg.	*d* = 0	*d* = 0	*d* = 0
NTBD_Payout_	0.2537*** (0.0874)	0.2960*** (0.0588)	0.3779*** (0.0579)	− 0.0063 (0.0888)	0.0638 (0.0695)	0.1398** (0.0565)
OWN	− 0.0250* (0.0149)	− 0.0499*** (0.0127)	− 0.0350** (0.0141)	− 0.0256* (0.0137)	− 0.0478*** (0.0122)	− 0.0351*** (0.0136)
NTBD_Payout_·OWN	0.3155*** (0.1034)	0.3815*** (0.0946)	0.4511*** (0.0920)	0.3708*** (0.1011)	0.4195*** (0.0856)	0.5082*** (0.0900)
Firm-controls	Yes	Yes	Yes	Yes	Yes	Yes
Country-controls	Yes	Yes	Yes	Yes	Yes	Yes
Firm-FE	Yes	Yes	Yes	Yes	Yes	Yes
Year-FE	Yes	Yes	Yes	Yes	Yes	Yes
*N*	42,721	42,721	42,721	42,721	42,721	42,721
*R* ^2^	0.7274	0.7275	0.7274	0.7248	0.7247	0.7248
Combined effect (= NTBD_Payout_ + NTBD_Payout_·OWN)	0.5692	0.6775	0.829	0.3645	0.4833	0.6480

This table shows regression results for six different measures of the firm-specific dividend payout ratio, *d*. In all regressions, firm-level as well as country-level control variables from Table 7 as well as firm- and year-fixed effects are included, but not reported. Robust standard errors, clustered at country-level, are presented in parentheses

***, **, and * indicate statistical significance at the 1%, 5%, and 10% levels, respectively

Throughout our robustness checks, the effect of NTBD_Payout_ on debt ratios increases in the level of individual ownership of the firm, although the magnitude of the combined effects varies with respect to the calculation of *d*. Our main finding from Sect. 4 that the effect for firms that are wholly owned by an individual is about twice the effect for firms with an individual owner as the largest owner holds for all alternative definitions of *d*.^14^

In another robustness test, we address the problem of calculating firm-specific dividend payout ratios for years in which the increase in shareholder funds from *t* − 1 to *t* is larger than the observed profit in *t*. In this case, some changes in shareholder funds must be driven by changes in reserves that we cannot observe directly from our data. In our sample, nearly 33% of all firm-years show changes in shareholder funds that are larger than the observed positive profit. So far, we assume no dividend payments for those years. In a robustness test, we eliminate all firm-years for which the increase in shareholder funds from *t* − 1 to *t* is larger than the observed profit in *t*. This reduces our sample size to 28,634 firm-year observations. Excluding those years from the analysis raises the average firm-specific dividend payout ratio from 0.3235 to 0.4012, whereas the average firm-specific net tax benefit of debt rises only marginally from 0.1294 to 0.1362. However, results in columns (1a) to (1c) in Table 13 show that the main results from Table 10 still hold.

**Table 13:** Robustness tests, 2002–2012

Variables	Specification					
	(1a)	(1b)	(1c)	(2a)	(2b)	(2c)
	*OWN *=* LargeOwn*	*OWN *=* MajOwn*	*OWN *=* WhollyOwn*	*OWN *=* LargeOwn*	*OWN *=* MajOwn*	*OWN *=* WhollyOwn*
NTBD_Payout_	− 0.0915 (0.1128)	0.0006 (0.0714)	0.0180 (0.0588)	− 0.1395 (0.0939)	− 0.0578 (0.0574)	0.0085 (0.0464)
OWN	− 0.0119 (0.0189)	− 0.0205 (0.0137)	− 0.0073 (0.0158)	− 0.0270* (0.0161)	− 0.0368*** (0.0123)	− 0.0416*** (0.0150)
NTBD_Payout_·OWN	0.2635** (0.1183)	0.2305*** (0.0851)	0.3609*** (0.0977)	0.3522*** (0.0992)	0.3282*** (0.0817)	0.4526*** (0.0952)
Firm-controls	Yes	Yes	Yes	Yes	Yes	Yes
Country-controls	Yes	Yes	Yes	Yes	Yes	Yes
Firm-FE	Yes	Yes	Yes	Yes	Yes	Yes
Year-FE	Yes	Yes	Yes	Yes	Yes	Yes
*N*	28,634	28,634	28,634	22,859	22,859	22,859
*R* ^2^	0.7248	0.7247	0.7248	0.5762	0.5761	0.5762
Combined effect (= NTBD_Payout_ + NTBD_Payout_·OWN)	0.3178	0.4489	0.6183	0.3269	0.4456	0.6132

This table shows regression results for two further robustness tests. In specification (1) we exclude all firm-years for which the increase in shareholder funds from *t* − 1 to *t* is larger than the observed profit in *t*. In specification (2) we exclude all firms from Russia. In all regressions, firm-level as well as country-level control variables from Table 7 as well as firm- and year-fixed effects are included, but not reported. Robust standard errors, clustered at country-level, are presented in parentheses

***, **, and * indicate statistical significance at the 1%, 5%, and 10% levels, respectively

Russian firms make up about 47% of all of our sample companies and show the highest average debt ratio among all sample countries. We therefore exclude 19,862 firm-year observations for Russian firms from our sample. The main results shown in column (2a) to (2c) in Table 13 still hold.^15^

Throughout our robustness tests we are able to show that the influence of the net tax benefit of debt on debt ratios is higher the higher the level of firm-specific individual ownership. Also, we are able to show that jointly considering firm-specific dividend payout policy and firm-specific ownership reduces measurement error, as the combined effects found in Tables 12 and 13 are always larger than the baseline effect for NTBD in column (1) in Table 9 (0.2680).

## Conclusion

Interest payments for debt are tax-deductible at the corporate level which creates an interest tax-shield, while payments to equity investors are not. This causes a tax distortion to firm behavior, as debt becomes relatively more attractive than equity. Following Miller (1977), several papers have shown that not only corporate, but also investor level taxes have to be considered when measuring the net tax benefit of debt due to the so-called personal tax penalty. In this paper, we add to the literature by jointly analyzing the influence of investor level taxes, firm-specific ownership and firm-specific dividend payout policy to provide a more precise measure of the effect of taxes on capital structure choice.

We follow the definition of the net tax benefit of debt by Gordon and MacKie-Mason (1990) and use data from 11 countries within Central and Eastern Europe over the period 2002–2012 to test whether higher net tax benefits of debt result in higher debt levels. Contrary to prior research, we can observe firm-specific dividend payout ratios and the firm-specific ownership structure on a yearly basis. This allows us to identify whether a firm has an individual marginal owner in a given year and to test the effect of the net tax benefit of debt on debt levels with respect to different definitions of the marginal owner.

In line with prior research, we find that debt ratios in our sample increase with the net tax benefit of debt. Not considering firm heterogeneity, an increase in the net tax benefit of debt of 10 percentage points leads to an increase in debt ratios of 2.68 percentage points. Our sample firms distribute, on average, only a third of their profits as dividends, whereas the rest of the profits are retained and subject to capital gains taxation in the future. We show that considering firm-specific dividend payout policy, without controlling for firm-specific ownership, leads to an underestimation of the effect of the net tax benefit of debt on debt levels. The more precise measurement of the net tax benefit of debt, including firm-specific dividend payout policy, is outweighed by the measurement error due to ignoring firm-specific ownership.

We show that it is important to simultaneously control for both sources of firm heterogeneity when evaluating the effect of investor level taxes on debt ratios, especially if the firm has an individual as the majority owner or is wholly owned by an individual. The size of the effect of the net tax benefit of debt on debt ratios crucially depends upon the definition of the marginal owner of the firm. It is highest if the firm is wholly owned by an individual. In this case, the effect of the net tax benefit of debt on debt ratios is about twice the effect when controlling for neither firm-specific dividend payout policy nor firm-specific ownership.

Our results are robust to different measures of the net tax benefit of debt, i.e., different calculations of the dividend payout ratio as well as the effective tax rate on capital gains. Our findings add to prior literature by providing a more precise measure of the effect of investor level taxes on capital structure choice. We can show that it is important to consider the interplay of both sources of firm heterogeneity (ownership and dividend payout policy), rather than separately controlling for them. We also show that ignoring firm heterogeneity can lead to a severe underestimation of tax effects. Using owner-specific tax rates that are based on firm-specific ownership information is crucial in determining the effect of taxes on debt ratios, especially if ownership is less dispersed.

Of course, our study is subject to several limitations. Data restrictions, such as measurement of the payout ratio, are due to data availability in the relevant databases and can only be avoided by hand-collecting information from prohibitively large numbers of financial statements. For similar reasons, hybrid financial instruments that can be qualified as either debt or equity cannot always be properly identified. Moreover, the qualification of provisions as debt is ambiguous across jurisdictions. Although we have detailed annual ownership data, we cannot observe whether or not share capital and voting rights are equivalent. It is therefore possible that preferential voting rights enable minority shareholders to dominate a corporation and to enforce their favorite debt policy. Furthermore, tax rulings such as tax holidays for particular investors can distort the net tax benefit of debt. Since tax rulings are not publicly observable,^16^ our study relies solely upon statutory corporate and individual tax rates.

There are several interesting avenues for future research regarding firm heterogeneity and capital structure. Apart from industry-related effects, a related test could be whether firms that were subject to major ownership changes also changed their capital structure and dividend policy. Such a result seems especially likely in cases of leveraged buyouts or management buyouts. Moreover, the impact of the tax sensitivity of leading individual shareholders on capital structure could be investigated in more detail by use of data from reported insider trades. However, this is only feasible for listed corporations, a small minority of all enterprises in Europe. For a more comprehensive view of corporate debt policy, it would be desirable to include leasing as a substitute for debt in our analysis. This extension, however, would require a detailed analysis of IFRS financial statements that are typically unavailable in databases. Similar data restrictions apply for hybrid financing that can be used as a device for tax avoidance.

## Footnotes

1 Due to the lack of information about the tax status of foreign owners, we focus on individual domestic investors only. Babbel et al. (2018) calculate the net tax benefit of debt for different owner types (i.e., foreign owners, corporate owners) using specific tax rates per owner type.

2 The countries in our sample are former transition economies. As Klapper et al. (2002) point out, these economies differ from market economies, i.e., the US or Western Europe in economic aspects such as a concentration of firms in the industrial and manufacturing sector, the underdevelopment of financial systems (inability to borrow long-term caused by inflation and weak legal protection) and low legal and governance standards. From an institutional point of view, most firms in CEE countries are organized (and taxed) as corporations rather than partnerships and individual ownership is very high in CEE firms and individual domestic owners are typically the largest owner group. Therefore, special attention has to be given to the question whether traditional capital structure theories, as assumed by the model of Gordon and MacKie-Mason (1990), can be applied to CEE firms. Results from Klapper et al. (2002), Berk (2006) and Delcoure (2007) show that both the trade-off theory and the pecking order theory can explain capital structure choices in the CEE countries included in our analysis. In addition, the relevance of corporations compared to partnerships and sole proprietorships is higher in the CEE countries than for the EU average. Whereas 34.6% of active EU firms in the business economy were limited liability enterprises in 2012, the corresponding percentages are much higher in most of our sample countries (Bulgaria 66.2%, Estonia 83.3%, Croatia 60.4%, Latvia 74.3%, Lithuania 43.2%, Hungary 45.3%, Romania 65.7%. Only Poland with 7.1% and Slovakia with 30.8% had lower percentages). A similar relation applies to the number of employees in the population of active enterprises. In 2012, 63.8% of employees in the business economy were employed by limited liability enterprises in the EU. The corresponding percentages in our sample countries are: Bulgaria 85.0%, Estonia 94.7%, Croatia 82.5%, Latvia 81.3%, Lithuania 86.4%, Hungary 60.6%, Poland 48.5% Romania 93.0%, Slovakia 70.4%. See Eurostat (2018a). With respect to firm size, the average total assets for about 2.2 million European firms with data available in AMADEUS in 2012 is 29,716 thd Euros, compared to 46,432 thd Euros for our sample firms. Although corporations tend to be the most important legal form in the CEE countries, this applies only to private corporations. On average, the number of listed firms as well as the market capitalization tend to be low in our sample countries. Whereas equity market capitalization often exceeds 100% of GDP in major OECD countries, this value is typically far below 50% of GDP for our sample countries. See World Federation of Exchanges (2018). This result also indicates that CEE corporations are on average much smaller than other EU corporations and have much more restricted access to capital markets. Therefore, the shareholders of our sample firms often cannot simply take a loan on their shares or sell them. Another difference refers to the interest rates in the CEE countries that are/were not members of the Euro area. Interest rates for non-Euro country-years are (in some cases substantially) higher than for Euro country-years. See Eurostat (2018b).

3 This number is in line with data on international tax rates, e.g., Jacob and Jacob (2014) have shown that 11 of 25 countries applied different tax rates on dividends and capital gains in 2008.

4 We define firms with incomplete ownership information as firms for which the sum of the percentage of all owner blocks does not add up to at least 99%.

5 The same measure is used by several previous studies, e.g., Overesch and Voeller (2010), Pfaffermayr et al. (2013) and Babbel et al. (2018). AMADEUS does not provide information on the level of interest-bearing debt, as used by Faccio and Xu (2015).

6 We use different definitions and variations of the firm-specific dividend payout ratio in our robustness tests in Sect. 5. Some of them address the problem that the AMADEUS database does not permit us to separately observe changes in reserves.

7 These numbers are in line with international data as well as data for listed CEE firms. Over the observation period 2002–2012 the average annual dividend payout ratio of S&P 500 firms varied between 25% and 38%. At the same time, the average annual dividend payout ratio of the Stoxx Europe 600 firms was slightly higher, varying between 42% and 53%. Lace et al. (2013) analyze dividend payout ratios of CEE listed firms and find average dividend payout ratios of 30%.

8 If, for example, the holding of the largest individual owner accounts for 35%, we classify a firm as having an individual owner as its largest owner, as long as there is no other owner who holds more than 35% of the firm. However, we do not classify this firm as having an individual owner as its majority owner (to be wholly owned by the individual owner), as the percentage of ownership is below 50% (100%). Thus, for such a firm, *LargeOwn* is 1, but *MajOwn* and *WhollyOwn* are both 0.

9 The tax status of an individual shareholder as measured by the variable OWN does not necessarily correspond to the tax status of the creditors of the firm, who might be different individuals or corporations. However, the comparison of the Miller (1977) index refers to the decision of a given individual rather than different financiers between providing debt or equity. The interaction coefficients of OWN and investor level tax rates ($\tau_{d}$, $\tau_{g}$, and $\tau_{i}$) show the effect of investor level taxes for firms with a tax-sensitive marginal investor. Only for a tax-sensitive marginal investor, we expect a negative relation between the personal interest tax rate and the debt ratio of a firm, because a tax-sensitive marginal investor would also have been able to provide debt instead of equity.

10 In additional specifications, we add the interaction of *NoDivPayer* and the capital gains tax rate as well as the triple interaction with firm-specific ownership variables to the estimation. Non-tabulated results show a significant positive effect of all interaction terms including capital gains taxes, indicating that the effect of capital gains taxes on capital structure is higher for non-dividend paying firms.

11 In comparison, the coefficient of Overesch and Voeller (2010), based on a sample of European countries, is 0.2870, and the coefficient for Faccio and Xu (2015) for a sample of OECD countries is 0.201, showing that the effects found for CEE countries do not differ substantially from other countries.

12 Among our sample countries we find lower tax rates on dividends than on capital gains for only 29 out of 121 country-year observations (23.96%).

13 If the firm-specific average dividend payout ratio is negative, we assume a payout ratio of 0 and if the firm-specific average dividend payout ratio is above 1, we assume a payout ratio of 1.

14 Comparing the combined effects in Table 12 to those in Table 9 shows higher combined effects, if we use average dividend payout ratios rather than firm-year specific dividend payout ratios for the calculation of the net tax benefit of debt. This result might be an indicator that the firm-year specific dividend ratio is an imprecise measure of the dividend strategy of the firm. As a consequence, average payout ratios over a longer period, rather than single-year dividend payments, should be used to calculate the net tax benefit of debt.

15 In another robustness test, we exclude firms from Estonia, Latvia and Slovakia from our sample, as the average net tax benefit of debt is negative (Estonia) or close to zero (Latvia and Slovakia) for these countries. Non-tabulated results for a sample of 40,605 firm-year observations show that our main results still hold.

16 The so-called LuxLeaks are an example for involuntary publication of confidential tax data such as tax rulings. See International Consortium of Investigative Journalists (2016).
